# Temperature-Dependent Development and Survival of Brazilian Populations of the Mediterranean Fruit Fly, *Ceratitis capitata*, from Tropical, Subtropical and Temperate Regions

**DOI:** 10.1673/031.012.3301

**Published:** 2012-03-02

**Authors:** Marcelo P. Ricalde, Dori E. Nava, Alci E. Loeck, Michele G. Donatti

**Affiliations:** ^1^ Departamento de Fitossanidade, FAEM/UFPel, Cx. Postal 354, 96010-900, Pelotas, RS, Brasil; ^2^ Laboratório de Entomologia, Embrapa Clima Temperado, Rodovia Br 396, km 78 Cx. Postal 403, 96001-970, Pelotas, RS, Brasil

**Keywords:** fruit pests, medfly, Mediterranean fruit fly, thermal requirements

## Abstract

The Mediterranean fruit fly *Ceratitis capitata* (Wiedemann) (Diptera: Tephritidae) is one of the principal exotic pests affecting Brazilian production in the northeastern and southeastern regions of Brazil. In the south, it is has potential as a serious threat to temperate-climate fruit farms, since it is already found in urban and suburban communities in this region. We studied the biological characteristics of *C. capitata* populations from Pelotas-RS (temperate climate), Petrolina-PE (tropical), and Campinas-SP (subtropical). *Ceratitis capitata* biology was studied under controlled temperature (15, 20, 25, 30, and 35 ± 1 ^°^C), 70 ± 10% RH, and 14:10 L:D photoperiod. The duration and survival rate of the egg, larval, and pupal stages were evaluated and the thermal requirements of these three populations were determined. The duration and survival of these developmental stages varied with temperature, with similar values for the three populations, except for some variation in the egg phase. Egg to adult developmental time for all three populations was inversely proportional to temperature; from 15 to 30 ^°^C developmental time varied from 71.2 to 17.1, 70.2 to 17.1, and 68.5 to 16.9 days, respectively. Survival during development was affected at 15 to 30 ^°^C, and differed significantly from survival at 20 to 25 ^°^C. At 35 ^°^C, immature stages did not develop. The basal temperature and degree-day requirement were similar for all immature stages except for the egg stage. The basal temperatures and thermal constants were 9.30 and 350, 8.47 and 341, and 9.60 ^°^C and 328 degree-days for the Pelotas, Petrolina, and Campinas populations, respectively. Results suggested that survival and thermal requirements are similar for these tropical, subtropical, and temperate populations of *C. capitata*, and demonstrate the species' capacity to adapt to different climate conditions.

## Introduction

The Mediterranean fruit fly *Ceratitis capitata* (Wiedemann) (Diptera: Tephritidae) is the most important fruit fly pest of fruit production worldwide (White and ElsonHarris 1992). Its highly adaptable nature is one of the factors that has allowed it to become established throughout much of the world (Metcalf 1995). Additionally it is polyphagous, feeding on more than 200 different host species (Liquido et al. 1991).

Originating from tropical regions of Africa, *C. capitata* is also found in southern Europe, the Middle East, Central America, the Caribbean, Australia and parts of Oceania, and is distinctly absent in cold regions of the world (Malavasi et al. 2000). The first report of this species in Brazil was made by Ihering (1901). Until the 1980s, *C. capitata* was restricted to the southern and southeastern parts of the country, with the Recôncavo Baiano acting as its northern limit (Malavasi et al. 1980). Since then it has been found throughout the country, including the northeast (Morgante 1991; Feitosa et al. 2007) and the north (RonchiTelles and Silva 1996; Silva et al. 1998). In the state of Rio Grande do Sul, the Mediterranean fruit fly was found in urban areas of Pelotas and Porto Alegre in the 1960s (Bertels and Baucke 1966) and 1980s (Lorenzato 1984), respectively. This pest has not yet been reported in commercial peach and apple orchards (Salles and Kovaleski 1990), though it was found in suburban areas of Pelotas near commercial peach orchards, especially in guava *Psidium guajava* and persimmon *Dyospirus kaki* (Nava et al. 2008).

Introduction of the Mediterranean fruit fly to the various regions of the world has had a negative impact on fruit crops. In Brazil, it is estimated that this fruit fly pest causes production losses of 20 to 50% (Baldez 1972; Orlando and Sampaio 1973). As an example of its potential impact, if the USA state of California could not export fruit because of *C. capitata* infestation, there would be a loss of 35,000 jobs, 3.6 billion dollars in production loss, and a reduction in family income of 939 million dollars (Zucchi 2001).

Although it is found in all regions of the country, *C. capitata* has attained the status of a pest principally in southeastern Brazil, attacking mainly temperate fruit (Malavasi et al. 2000). The fact that it has become a pest in some regions and not in others could be due to regional climatic differences. Among climatic factors, temperature is the main ecological factor affecting insect growth and development, since it directly affects physiological processes and enzyme activity (Trudgill et al. 2005).

Insect development depends on thermal requirements. Each insect species has an optimal temperature range for development limited by lower and upper thresholds (base temperature (Tb) and upper limit (Ts)). Below and above these temperature limits, development does not occur (Haddad et al. 1999). In the range between Tb and Ts, insects accumulate degree-days and are able to develop. However, the thermal requirements of a species vary with developmental stage and geographic origin (Haddad et al. 1999; Honék and Kocourek 1990). According to Honék (1996), Tb tends to decrease with increasing latitude. Insect species that live in the tropics have a higher Tb (13.7 ^°^C) than those living in subtropical (10.5 ^°^C) or temperate regions (7.9 ^°^C).

Here we studied the effects of temperature on the biology of three Brazilian populations of *C. capitata* that had been collected from temperate, subtropical, and tropical regions to determine if they differed in their response to temperature during development.

## Materials and Methods

This work was carried out at the Entomology Laboratory of the Centro de Pesquisa Agropecuária de Clima Temperado (CPACT) - Embrapa Clima Temperado, Pelotas, Rio Grande do Sul. The three populations of *C. capitata* came from Pelotas, Rio Grande do Sul (RS) (31° 52′ S, 52° 21′ W, 13 m altitude; temperate climate), Petrolina, Pernambuco (PE) (9° 9′ S, 40° 22′ W, 400 m altitude, tropical climate) and Campinas, São Paulo (SP) (22° 53′ S, 47° 4′ W, 680 m altitude; subtropical climate). The temperature and rainfall conditions for these sites are given in Table 1. Temperate region flies were collected from persimmon *Diospyros kaki* Thunberg (Ericles: Ebenaceae), the tropical region flies from guava *Psidium guajava* L. (Myrtales: Myrtaceae), and the subtropical region flies from coffee *Coffea arabica* L. (Gentianales: Rubiaceae). The former of the two populations were collected from the wild, while the latter population came from a rearing facility maintained in the Entomology Laboratory of the Instituto Biológico de Campinas. This colony has been maintained since 1993, though wild flies from the same region are introduced periodically. Before the experiment, all three populations were reared in the laboratory for two generations on artificial diet using a rearing technique developed by Salles (1992) for rearing *Anastrepha fraterculus.*

We used climate chambers to maintain constant temperatures of 15, 20, 25, 30, and 35 ± 1 ^°^C, 70 ± 10% RH, and 14:10 L:D photoperiod. Duration, viability, and thermal requirements were determined for the egg, larval, and pupal stages and the egg-adult interval.

Artificial oviposition substrate was molded from blackberry gelatin using plastic molds. After the 250 mL artificial fruit medium became firm, it was wrapped in Parafilm (Bemis Company Inc., www.parafilm.com) and then offered to flies in rearing cages, where the females were allowed to lay eggs for ∼ 12 hours. The artificial oviposition substrate was then removed from the cages and placed in a 500 mL beaker and dissolved in warm water (∼ 40 ^°^C) using a Fisatom model 752A magnetic stirrer (www.fisatom.com.br), regulated to speed 8 for a duration of 10 min. The eggs were strained out and removed with a fine paintbrush and distributed on plastic Petri dishes (6.0 cm diameter × 1.5 cm deep), which were lined on the bottom with a moistened piece of filter paper. Twenty-five eggs were transferred to each Petri dish with five replicates, giving a total of 125 eggs for each temperature treatment. After the Petri dishes were sealed with PVC film to prevent the larvae from escaping, they were placed in climatic chambers.

Larval development was followed on an artificial medium made from brewer's yeast and wheat germ, using a technique developed by Salles (1992). After preparation, 200 mL of the medium was placed in plastic containers (10 cm high × 8 cm diameter). After it solidified, 25 larvae that were up to 12 hours old were transferred onto the medium. During the pupal stage, flies were maintained in plastic Petri dishes (10 cm diameter × 2.5 cm deep) containing moist vermiculite. Twenty-five pupae were used for each replicate, totaling 125 pupae for each temperature. The number of eclosed larvae, the number of new puparia, and the number of newly emerged adults for each temperature were recorded daily in order to determine the duration and viability of each developmental stage and to calculate thermal requirements.

**Table 1.**
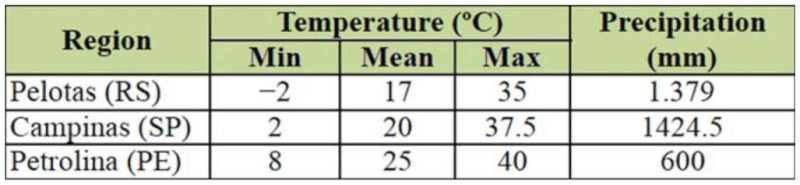
Minimum, mean, and maximum temperatures (°C) and mean monthly precipitation (mm) in the three climate regions from which the *Ceratitis capitata* populations were collected.

**Table 2.**
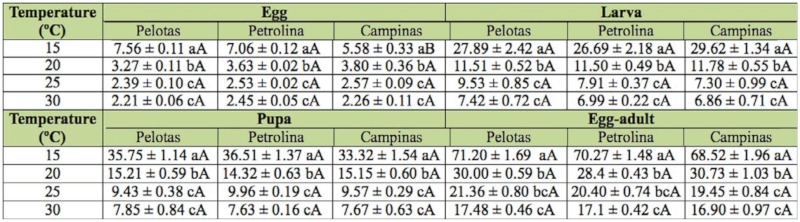
The duration of development (mean ± SE) (d) of the egg, larval, and pupal stages and egg-adult development period of populations of *Ceratitis capitata* from Pelotas-RS, Petrolina-PE, and Campinas-SP at various temperatures.

The experimental design was completely random, with five temperature treatments and five replicates, each consisting of 25 eggs, larvae, or pupae for the immature stages. The data on duration and viability were tested for normality using the Bartlett test. Data that was considered normally distributed was submitted to analysis of variance, and means were compared using the Tukey test with α = 0.05 (SAS Institute 2002).

**Table 3.**
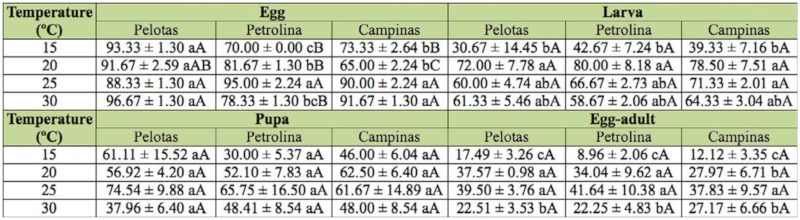
Viability (mean ± SE) (%) of the egg, larval, and pupal stages and egg-adult development period of *Ceratitis capitata* populations from Pelotas-RS, Petrolina-PE and Campinas-SP, at different temperatures.

Stage duration data at the different temperatures was used to calculate the lower thermal development threshold temperature (TT) and the thermal constant (K) by the hyperbolic method using MOBAE (Modelos Bioestatísticos Aplicados á Entomologia) (Haddad et al. 1999). The thermal requirements of the different populations of *C. capitata* were compared based on comparison of 95% confidence intervals according to Gangavalli and Aliniazee (1985). Based on thermal requirements, the potential number of generations of *C. capitata* per year was calculated for Pelotas-RS, Campinas-SP, and Petrolina-PE, taking into account the mean temperature data from the last 20 years from these locations. Calculation of the number of generations was made using the formula


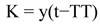


where K = number of degree-days, y = number of days necessary to complete the cycle, t = mean monthly temperature, and TT = threshold temperature of *C. capitata* determined in the laboratory.

## Results

The duration and survival of different stages of development and of the egg-adult interval of the three populations of *C. capitata* varied as a function of temperature (Tables 2 and 3). No development occurred at 35 ^°^C. The duration of the embryonic stage decreased with increasing temperature for all three populations, with significant differences between temperatures (*p* ≤ 0.05), except between 25 and 30 ^°^C. When egg developmental period was compared between populations, significant differences were only found at 15 ^°^C for the Campinas population compared to the other two populations *(p* ≤ 0.05, Table 2). Survival was higher than 88% for the Pelotas population, with no significant differences among temperatures (Table 3). We found that low temperatures were prejudicial for egg development in the other populations of *C capitata*, especially 15 ^°^C for the population from Petrolina and 15 and 20 ^°^C, for the population from Campinas, with eclosion taking significantly longer than at the other temperatures *(p* ≤ 0.05) (Table 3). When comparisons were made between populations at the same temperature, the highest survival was generally observed for the Pelotas population, except for at 25 ^°^C, at which there were no significant differences among populations, and at 30 ^°^C, at which the survival of the Pelotas population did not differ significantly from that of the Campinas population (Table 3).

The duration of the larval stage differed significantly across 15, 20, and 25 ^°^C for all three populations, though it did not differ significantly at 25 and 30 ^°^C *(p* ≤ 0.05) (Table 2). There were no significant differences among populations in larval stage duration at the same temperature (Table 2). Survival during the larval stage was highest at 20, 25, and 30 ^°^C for all three populations, although larval survival at 25 and 30 ^°^C for the Petrolina and Pelotas populations and at 30 °C for the Campinas population did not differ significantly from the values obtained at 15 ^°^C (Table 3). Survival at the same temperature did not vary significantly among populations (Table 3).

Similar to what was found for the egg and larval stages, the duration of the pupal stage was inversely proportional to temperature (Table 2). The duration of the pupal stage at 15, 20, and 25 ^°^C differed significantly among these temperatures for all three populations of *C. capitata*, though the duration at 25 ^°^C did not differ significantly from values being observed at 30 ^°^C (Table 2). The duration of the pupal stage at the same temperature did not differ significantly among the three populations, nor did survival of the pupal stage differ across temperatures or populations (Table 3).

Total developmental time differed significantly at the different temperatures, except at 25 ^°^C, which did not differ from the duration at 30 ^°^C (Table 2), but did not differ significantly among populations when compared at the same temperature. Egg to adult viability of the three populations at 20 and 25 ^°^C differed significantly from survival at 15 and 30 ^°^C; survival also differed significantly between the latter temperature extremes (Table 3). No significant differences in egg-adult viability were found between the three populations at the same temperature (Table 3).

**Table 4.**
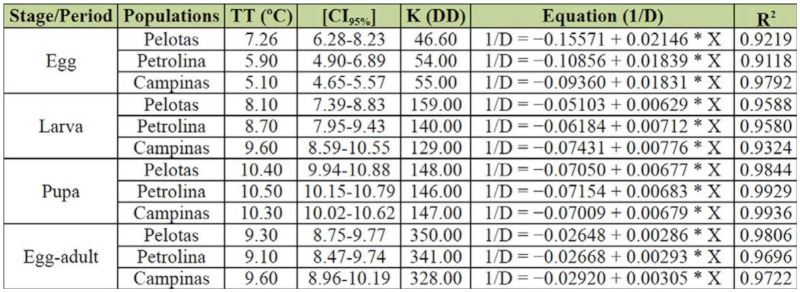
Threshold temperature (TT), confidence interval (CI), thermal constant (K), development equation (I/D), and determination coefficient (R^2^) for the egg, larval and pupal stages and the egg-adult development period of *Ceratitis capitata*, from different localities.

The threshold temperature for egg development in the Pelotas population (7.26 °C) was significantly different from that of the Campinas population (5.10 ^°^C), since the 95% confidence intervals did not overlap, whereas the threshold temperature (TT) for the Petrolina population (5.9 ^°^C) did not differ significantly from the others (Table 4). For the embryonic period and the other developmental stages, the R^2^ was always above 91% (Table 4), being therefore superior to the 90% predicted by the hyperbole method (Haddad et al. 1999).

Larval stages of *C. capitata* from the Pelotas, Petrolina, and Campinas populations had a threshold temperature and a thermal constant (K) of 8.1 and 159.0, 8.7 and 140.0, and 9.6 ^°^C and 129.0 degree-days, respectively, and for the pupal stage had 10.4 and 148, 10.5 and 146, and 10.3 ^°^C and 147 degree-days, respectively (Table 4).

Total developmental time of *C. capitata* from Pelotas, Petrolina, and Campinas required a threshold temperature and a K of 9.3 and 350, 9.1 and 341, and 9.6 ^°^C and 328 degree-days, respectively, indicating that for complete development (egg to adult) the thermal requirements of the three populations were similar (Table 4).

Based on these thermal requirements of the egg-adult period, it was estimated that *C. capitata* could reach 9.04, 14.18, and 17.90 generations per year in Pelotas, Campinas, and Petrolina, respectively.

## Discussion

The data obtained in this study indicate that *C. capitata* from a temperate climate zone (Pelotas-RS) have the same thermal requirements as populations from sub-tropical Campinas-SP and tropical Petrolina-PB, and do not support the hypothesis that populations from different climatic regions should differ in their thermal requirements.

The three populations of *C. capitata* developed at all temperatures except 35 ^°^C. The duration of the embryonic period of these populations at 15 and 30 ^°^C was similar to those reported by Duyck and Quilici (2002), who observed 7.79 to 1.54 days at these temperatures. They also found that greater than 81% of eggs hatched, which is comparable to values found in our study.

The larval stage durations found in our results were longer than reported by Duyck and Quilici (2002), who reported values of 21, 8, 6, and 5 days at 15, 20, 25, and 30 ^°^C, respectively, and similar to those observed by Grout and Stoltz (2007) at 14 and 30 ^°^C (31.6 and 6.2 days, respectively). One factor that likely accounts for some differences in developmental time was the different larval diets. While our diet was mainly wheat germ and brewer's yeast, Duyck and Quilici (2002) used wheat germ and carrot powder. Protein sources are important for *C. capitata* because they supply essential amino acids; deficiency during the immature stage can increase the larval stage duration (Cangussu and Zucoloto 1997). We observed survival rates from 20 to 30 ^°^C that ranged from 80 to 68%, which was lower than the survival rates found for the *C. capitata* population from Reunion Island that had a viability above 80%, but similar to a population from South Africa that was above 70% at 18–30 ^°^C (Grout and Stoltz 2007). At 15 ^°^C, larval survival was below 42%, demonstrating that low temperature is prejudicial for *C. capitata* development (Duyck and Quilici 2002), and a similar value was reported by Grout and Stoltz (2007) at 14 ^°^C (43.7%). The fact that differences were not found in duration and viability between the populations when measured at the same temperature indicates that these three populations of *C. capitata* collected from different localities in Brazil are biologically similar in this regard, although *C. capitata* strains can be differentiated (Douglas and Haymer 2001). The fact that the Mediterranean fruit fly developed at nearly all of these temperatures with moderate to high survival illustrates one reason this species is a cosmopolitan pest capable of surviving a wide range of temperatures that prevail from the northern part of Brazil (tropical climate) to the southern part (subtropical and temperate climates).

The duration of the pupal stage of our *C. capitata* populations was similar to values reported by Duyck and Quilici (2002), who recorded 35, 17, 10, and 8 days at 15, 20, 25, and 30 ^°^C, respectively, and the values obtained by Grout and Stoltz (2007) 31.2, 20.0, 13.1, 8.9, and 9.1 days at 14, 18, 22, 26, and 30 ^°^C, respectively. However, Duyck and Quilici (2002) and Grout and Stoltz (2007) reported survival rates above 75%, which were much higher than ours. In our study, pupae were left in contact with moist vermiculite and may have become contaminated with bacteria or fungi.

Total developmental time for *C. capitata* from Pelotas, Petrolina, and Campinas was slightly longer at 15 ^°^C than that reported for flies from the Reunion Islands (64 days), whereas at other temperatures the durations were similar (Duyck and Quilici 2002). As the eggadult interval is the sum of the intervals for all developmental stages, it is observed that the differences compared to Duyck and Quilici (2002) are largely due to differences in duration of the larval stage, when food quality directly affects developmental time and viability (Zucoloto 2000).

Among all the developmental stages, the embryo best tolerates low temperatures; this appears to be a general rule for tephritids (Salles 1992). The threshold temperature values found for the embryonic period of *C. capitata* of the three populations were lower than found for populations from Reunion Island (11.6 ^°^C) (Duyck and Quilici 2002) and South Africa (9.6 ^°^C) (Grout and Stoltz 2007). Messenger and Flitters (1958) also reported a threshold temperature of 11.7 ^°^C and a thermal constant of 25.74 degree-days. In contrast, Fares (1973) found a threshold temperature of 9.7 ^°^C, similar to what we found for *C. capitata* in Brazil. There is considerable variation in base temperature from different reports, probably due to the geographic origin of these populations (Honék 1996).

The larval stage of *C. capitata* populations from Pelotas, Petrolina, and Campinas had a base temperature and a thermal constant of 8.1 and 159, 8.7 and 140, 9.6 ^°^C and 129 degreedays, respectively. Similar values were found for *C. capitata* from Reunion Island (TT = 10.2 ^°^C and K = 89 degree-days) (Duyck and Quilici 2002) and South Africa (TT =10.8 ^°^C and K = 94.5 degree-days) (Grout and Stoltz 2007). Lower values of TT (5.2 ^°^C) and K (139 degree-days) for *C. capitata* from Hawaii were reported by Vargas et al. (1996). Besides geographic origin, factors such as food quantity and quality and larval density in the rearing chambers can also influence the thermal requirements of the larval stage (Duyck and Quilici 2002).

The threshold temperature and thermal constant of the pupal stages were similar for the three populations. Similar base temperatures and thermal constants were reported by Duyck and Quilici (2002) (11.2 °C and 143 degree-days) and by Grout and Stoltz (2007) (9.4 ^°^C and 155 degree-days), and higher values were reported by Shoukry and Hafez (1979) (13 ^°^C). Fletcher (1989) indicated that the large differences in thermal requirements among various studies are mainly due to different methodologies and possibly because different strains of *C. capitata* were tested.

The threshold temperature and thermal constant for complete development of the three *C. capitata* populations were close to the values reported by Grout and Stoltz (2007) (9.9 ^°^C and 337.8 degree-days). Results suggest that the thermal requirements for the egg-adult period are similar for the three *C. capitata* populations, despite the fact that the Tb of the egg stage of the Pelotas population differs from that of Campinas. Also, based on the thermal classification of Honék (1996), these three populations can be classified as being subtropical, having a mean lower thermal development threshold of 10.5 ^°^C, which explains in part the fact that *C. capitata* is a key pest in the southeast region of Brazil.

Although the number of *C. capitata* generations is close to nine for Pelotas in the southern region of Brazil, during winter the population is small and there are no infestations during the cold period. In Campinas-SP in the southeast and PetrolinaPE in the northeast, *C. capitata* is active throughout the year and is considered one of the main pests of fruit crops (Malavasi et al. 2000). Though temperature is one of the main factors that affects the development of Mediterranean fruit fly, host availability is also a key factor for population increase and host fruits are frequent and abundant throughout the year in the southeast and northeast regions of Brazil.

We conclude that the *C. capitata* population in Pelotas has not yet impacted peach groves because it is still restricted to urban and suburban areas (Nava et al. 2008), though it has the potential to cause serious economic losses as it presently occurs in the southeastern and northeastern regions of this country. Consequently, the monitoring of fruit flies in peach orchards in the south should include not only *A. fraterculus*, which is attracted to hydrolyzed protein, but also should consider *C. capitata*, which can be attracted with sex pheromones.

## **Abbreviations: **

TT: threshold temperature;
K: thermal constant;
UA
